# AI Can Predict, but Experiments Must Test: A New Role for Neutron Facilities in a Materials AI World

**DOI:** 10.1021/acs.jpclett.6c02724

**Published:** 2026-09-24

**Authors:** Jeff Armstrong

**Affiliations:** † ISIS Neutron and Muon Source, Rutherford Appleton Laboratory, Harwell Campus, Didcot OX11 0QX, United Kingdom; ‡ Department of Chemistry, University of Bath, Bath BA2 7AY, United Kingdom

## Abstract

Materials AI is advancing at an exceptional pace and attracting growing investment, while the experimental scientific facilities and people needed to test and improve its predictions face mounting pressure. Unlike increasingly standardized data-center builds, neutron facilities are bespoke, source- and instrument-specific capabilities that take many years to build and mature, making lost capacity exceptionally difficult to replace. The response should be to embrace this shift by aligning experimental capability with the problems AI is increasingly being used to solve. For atomistic materials modeling, machine-learned interatomic potentials (MLIPs) provide a natural focus, and we propose coordinated experimental validation at two scales. Workhorse campaigns would combine automated room-temperature diffraction and low-temperature inelastic neutron scattering across reusable libraries of hundreds of well-characterized samples, with observables predicted before beamtime and compared systematically with experiment. Targeted parametric studies would examine selected systems across temperature, pressure, adsorption and guest loading using quasielastic neutron scattering, total scattering and complementary diffraction and spectroscopy. Together, these measurements would expose broad performance trends and physical origins of failure, while separating development, diagnostic and blind challenge data for subsequent model improvement. Neutron facilities are not alternatives to AI investment; they are experimental infrastructure through which atomistic materials AI can be tested against reality.

## Prediction Is Outrunning Experimental Testing

Atomistic artificial intelligence has crossed an important threshold. Machine-learned interatomic potentials (MLIPs) can explore structures, follow atomic motion for far longer than direct electronic-structure calculations and make predictions across chemical spaces that no experimental program could examine exhaustively.^1^ This is a remarkable scientific opportunity. It is also creating an imbalance. Our ability to generate atomistic predictions is growing much faster than our capacity to test what those predictions mean for real materials. Investment is rapidly ramping up in AI, while the large-scale facilities needed to challenge and improve these models face intense financial pressure. This asymmetry matters strategically. Computing capacity can be increased through increasingly standardized, modular data-centre builds. Neutron capability is not reproduced from a template: source architecture, moderators, shielding, beam transport and the instrument suite are tightly coupled to facility-specific design choices, and the resulting scientific capability matures over many years of operation. Those facilities are also more than machines. They are organically formed communities of scientists, engineers, technicians and users that have built decades of unique expertise. Closing such a facility can therefore remove a scientific capability that is far slower and more difficult to recreate than additional computing infrastructure. Investing in them is not an alternative to investing in AI. It is what keeps AI answerable to the physical world. The argument here is deliberately narrower than materials AI as a whole. Materials AI also includes generative models that propose new compositions or structures, surrogate models that predict electronic, optical, electrochemical or mechanical properties, and models that interpret experimental images or spectra. Those approaches require experimental tests matched to the claims they make: proposed structures can be challenged through synthesis and diffraction, predicted electronic or optical properties through transport or spectroscopy, and predicted functional performance through catalytic, electrochemical or mechanical measurements. Here we focus on MLIPs and related atomistic models because their predictions of equilibrium structure and atomic motion can be forward-modeled particularly directly into diffraction and neutron-scattering observables. The broader principle is the same: an AI prediction becomes physically persuasive when it is confronted with an independent measurement of the quantity, or a closely connected observable, that the model claims to describe.

The problem is not that MLIPs lack computational tests. Their energies, forces and stresses are normally evaluated against configurations withheld from training. These held-out configurations act like unseen examination questions and show whether a model can reproduce patterns beyond the examples used to fit it. Yet the questions and answers still come from the same computational teacher, usually density functional theory (DFT). Strong performance therefore establishes that an MLIP reproduces its chosen electronic-structure method over the configurations examined. This is of course an essential part of the process of developing accurate models, but it does not establish that either the teacher or the MLIP reproduces the real material.

This distinction becomes decisive because the real scientific question is rarely whether the average numerical error in the predicted forces is acceptably small. What matters is whether the model correctly predicts a phase boundary or diffusion coefficient, or captures the physical mechanism underlying adsorption or a chemical reaction. An interatomic potential is a mathematical landscape that assigns an energy to every possible arrangement of the atoms. Forces point down the slopes of that landscape, and a molecular-dynamics simulation follows them through time. No training set can cover the whole terrain. It may represent ordinary structures near equilibrium extremely well while barely sampling surfaces, defects, transition states or unusually short contacts. Errors may also be systematic, consistently making bonds too soft or barriers too low, and small force errors can accumulate during a long trajectory.

Most importantly, the property of interest may depend on a tiny and poorly sampled region. A vibrational frequency depends on the curvature around a local minimum, diffusion on the barrier between neighboring sites and a reaction rate on a narrow transition-state pathway. An average test error blends many configurations together and is often dominated by the easy cases. It can therefore conceal a rare local failure that controls the measured property, correlated errors that drift in the same direction, or an instability that appears only after an extended simulation. A model can achieve an excellent average score while getting the decisive piece of physics wrong.^2,3^

Recent benchmarks show that this concern is real rather than hypothetical. Matbench Discovery demonstrates that accurate formation energies do not necessarily lead to reliable identification of stable materials.^4^ Universal MLIPs can make materials systematically too soft, affecting surfaces, defects, migration barriers and phonons,^5^ while MS25 shows that low energy and force errors do not guarantee accurate volumes, barriers or the derived properties researchers ultimately want to use.^6^ Most directly, UniFFBench reports a substantial gap when universal force fields are compared with measured structural and elastic information across more than 1,500 mineral systems.^7^ The lesson is not that one benchmark should replace all others. It is that the evidence must move closer to the physical claim.

## Confidence Must Be Earned in Layers

Figure 1 expresses that progression as a traffic-light system. Red represents validation against computational reference data. Amber is the first experimental layer, asking whether the model reproduces the material at defined equilibrium or near-equilibrium state points. Green asks whether predictive agreement persists as controlled perturbations move the material beyond those reference states and through a wider region of its thermodynamic and configurational space. The colors are deliberately broad stages of increasing confidence rather than separate grades for every property a model might predict, and any designation remains bounded by the materials, conditions and perturbations actually explored.

**Figure 1:**
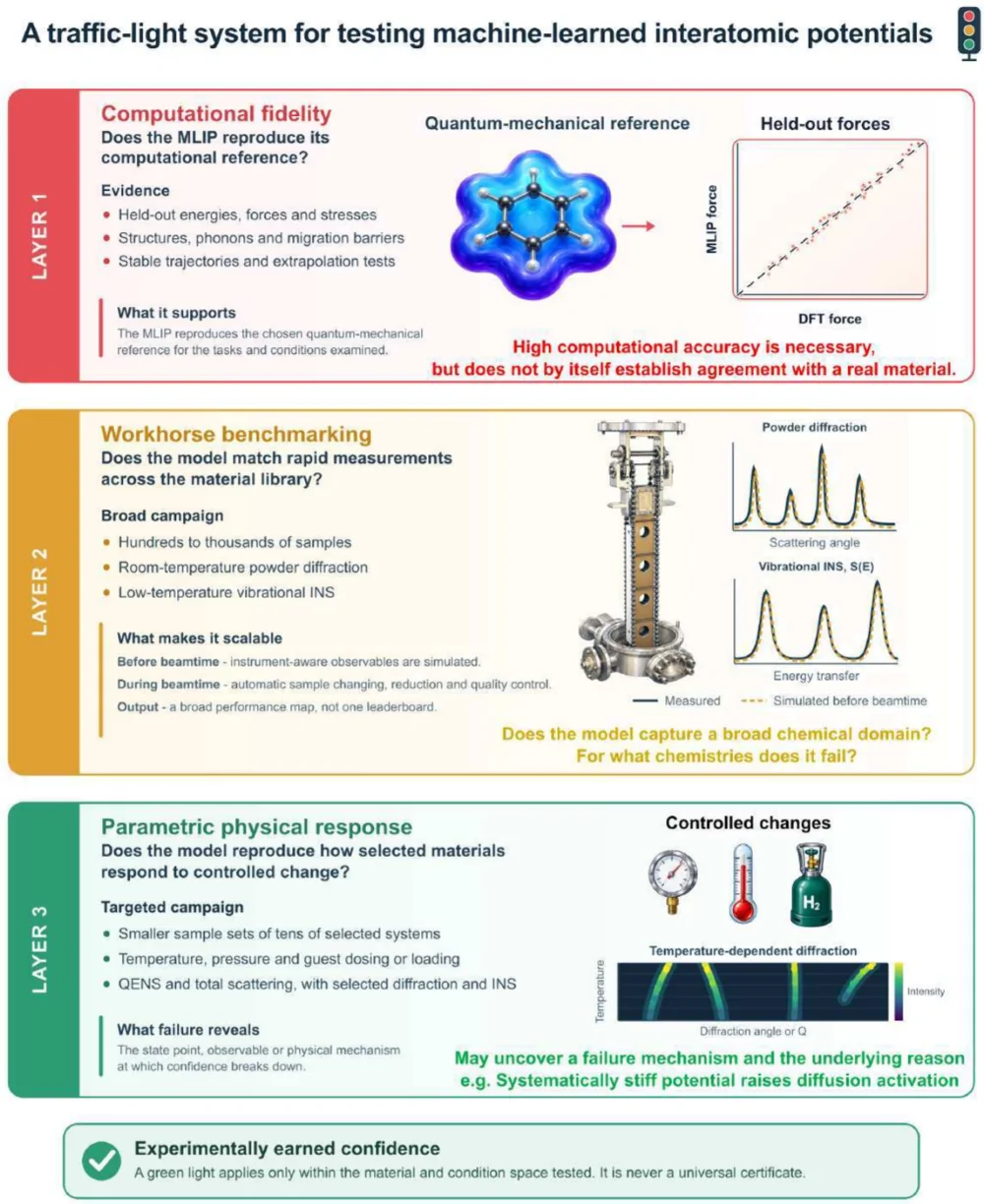
A traffic-light system for testing MLIP-based simulation workflows. Red establishes fidelity to computational reference data. Amber uses rapid workhorse measurements to test equilibrium or near-equilibrium structure and local energetics at defined state points. Green tests whether selected systems respond correctly as temperature, pressure, composition, adsorption, loading or another controlled variable is changed. The lights are a deliberately general hierarchy of progressively more demanding evidence; any confidence claim remains bounded by the materials, conditions and perturbations actually examined.

More specifically, the red layer asks how faithfully the MLIP has learned its computational teacher. Importantly, randomly splitting neighboring frames from one trajectory between training and testing can make the challenge look more independent than it is because adjacent configurations are often very similar. Stronger tests reserve an entire temperature range, phase, defect type, composition or deformation. Energies, forces and stresses should be examined across different chemical environments and energy ranges, alongside property-level tests such as structural relaxation, equations of state, phonons, migration barriers and stable molecular-dynamics trajectories. Committees of independently trained models can help identify unfamiliar configurations because disagreement between them often signals that the training data are sparse.^8–10^ Success at this layer means that the MLIP reproduces the chosen electronic-structure method for the tasks and conditions tested.

The amber layer moves from computational reference to measured reality. Its natural role is broad experimental benchmarking at defined equilibrium or near-equilibrium conditions. Diffraction can test the equilibrium atomic structure at one or more fixed state points, while low-temperature INS probes the local curvature of the potential-energy landscape around the occupied minimum. These are stringent physical tests, but they primarily establish whether the model reproduces the material within the region sampled by those measurements rather than how that description evolves under controlled perturbation. The comparison should be made in the language of the instrument. A forward calculation starts with the model’s predicted atomic arrangements or trajectories and calculates the diffraction pattern or spectrum those atoms would produce. Neutron or X-ray scattering weights, temperature, isotopic composition, statistical sampling and instrumental resolution must be included so that prediction and measurement can be compared like for like. Importantly, such a comparison tests a *model-and-simulation workflow*, not the MLIP alone. Sampling method, finite system size, treatment of temperature and anharmonicity, observable calculation and scattering approximations can all alter the predicted signal, while resolution, background, calibration and sample quality affect the measurement. Standards, repeats, uncertainty propagation and sensitivity tests on the simulation protocol should therefore accompany any strong claim of model failure. Flexible rescaling or indirect comparison through fitted structural parameters can otherwise hide a real discrepancy. Agreement with several complementary measurements is stronger than agreement with one because different techniques constrain different aspects of structure and motion.

The green layer asks whether the model-and-simulation workflow reproduces physical response as the system is deliberately perturbed beyond those equilibrium or near-equilibrium reference points. Temperature, pressure, composition, adsorption and guest loading can move a material into new structures and dynamical regimes. Matching several state points is more demanding than matching one because the workflow must reproduce the direction, scale and sometimes the abruptness of change. A shared offset may persist across every condition, whereas a wrong slope, transition point or change in diffusion exposes missing physics. The particular response being tested may be structural, thermodynamic or dynamical, and the traffic light is not intended to assign a separate color to each property. Rather, green records that the model has remained predictive across the controlled perturbations actually examined. Confidence therefore belongs to the region explored, not to the model in the abstract.

A rigorous campaign should separate experimental evidence into three roles. *Development data* may be used directly or indirectly to train, calibrate or refine a model. *Diagnostic benchmark data* can be used to compare models, identify failure classes and decide what should be improved. A third, genuinely *blind challenge set* should remain hidden from model development and selection: the participating groups would freeze model versions, simulation protocols and instrument-level predictions before the corresponding measurements are released. Training datasets and prior experimental calibration should be screened where possible so that apparent validation is not created by data leakage. Once a challenge measurement is used to guide refinement it becomes development or diagnostic evidence and should be replaced by a new held-out test for the next predictive claim.

Experiment and model development should therefore not be forced into opposition. When a prediction disagrees with a measurement, the natural scientific response is to localize the failure and improve the model. Experimental scattering data, differentiable trajectory reweighting and dynamical observables can help refine potential-energy surfaces beyond the limitations of a chosen DFT approximation.^11–13^ Neutron observables do not normally provide energies and forces directly, so the feedback loop is usually indirect: a structured discrepancy identifies the material, configuration space or interaction that is poorly described; targeted electronic-structure calculations are then generated for those configurations, training coverage is changed, or experimental observables are incorporated through reweighting or differentiable constraints. A pressure-dependent INS failure, for example, could motivate new reference calculations for compressed structures or intermolecular contacts implicated by the spectral shifts. New predictions must then be tested against fresh held-out measurements. This is the ordinary scientific cycle of prediction, measurement, learning and renewed prediction.

## Neutrons Provide a Distinctive Experimental Test

Large-scale neutron facilities are unusually well placed to make that cycle rigorous and scalable. Their value is not simply that they generate data. They bring independent measurement together with controlled sample environments, repeatable protocols, instrument modeling, research software, computing and shared expertise. Just as importantly, a facility is a living body of knowledge. A trustworthy result depends on instrument scientists who recognize artifacts, engineers who reproduce demanding environments safely, technicians who understand sample preparation and software specialists who preserve the path from raw counts to a physical result. Much of this judgement lives in experienced teams, not in manuals or archives. It cannot be mothballed and rapidly recreated when an AI model later needs an experimental answer.

Neutron scattering is particularly well matched to many of the structural and dynamical quantities that machine-learned interatomic potentials are built to describe. Diffraction reveals where atoms sit and how their positions are correlated, from the repeating crystal structure to local disorder, while neutron spectroscopy reveals how they move. Inelastic neutron spectroscopy (INS) resolves vibrations, whereas quasielastic neutron spectroscopy (QENS) follows slower processes such as molecular rotation, diffusion and jumps between sites. Together, these measurements span angstrom-to-nanometre distances and dynamical windows ranging from fast atomic vibrations to much slower molecular transport. These length and time scales directly overlap with those accessible to atomistic simulations driven by MLIPs. Neutron experiments can therefore test structures and motions that emerge from the model’s predicted atomic forces in an unusually direct way.

Neutrons are not a universal discriminator, however, and the strongest validation programs should use complementary measurements when they constrain different failure modes. X-ray scattering can provide rapid and highly precise structural information; infrared and Raman spectroscopy provide complementary vibrational selection rules; NMR probes local environments and motion; microscopy resolves microstructure and defects; calorimetry, transport and mechanical measurements test thermodynamic and functional response. The proposed neutron campaigns are therefore best viewed as one powerful component of a wider experimental validation ecosystem, with technique choice determined by the property the model is asked to predict.

## Breadth Needs Workhorses; Depth Needs Parametric Studies

Neutron techniques should not all be deployed in the same way, each having a distinct role in the assessment and development of MLIPs. We propose two complementary modes within one coordinated program. A workhorse campaign would sacrifice some environmental complexity in return for breadth, speed and reproducibility, targeting order 10^2^ unique, well-characterized physical samples under a small set of standard conditions; for sufficiently standardized measurements, a single coordinated campaign could realistically extend to several hundred samples. A targeted parametric campaign would concentrate much more beamtime on perhaps 5–20 carefully selected systems, following each across temperature, pressure, guest loading or another controlled perturbation. The workhorse campaign would reveal broad patterns of model performance, whereas the targeted campaign would ask whether the model captures the more nuanced behavior that often defines a material’s functionality. These strands can be planned together and run over the same program rather than being treated as successive rounds. Sample libraries and validation records reaching into the thousands are more realistically accumulated across repeated campaigns, facilities and model generations, particularly when a reusable physical library is revisited against new models or under new conditions.

The workhorse campaign should be genuinely industrial in organization, while remaining realistic about experimental throughput. Room-temperature powder diffraction and low-temperature INS are particularly well suited because measurements can be standardized, repeated rapidly and coupled to automatic sample changers. For an illustrative standardized measurement requiring roughly 0.5–2 instrument-hours per sample, a 100-sample library represents approximately 50–200 instrument-hours of counting before calibration, cryogenic and changeover overheads. The precise number is technique and sample dependent, but the order of magnitude makes a campaign of 10^2^ samples demanding yet credible and indicates how highly standardized programs could extend to several hundred samples; libraries approaching 10^3^ unique samples would more naturally be accumulated over multiple campaigns, cycles or facilities. Each physical sample would have a persistent identity linked to composition, synthesis, mass, container and quality-control information, and the same library should be retained and reused against later versions of the models wherever sample stability permits. Common cells and measurement times would reduce avoidable variation. Repeats, standards and empty containers would be built into the queue. The aim would not be to celebrate a handful of attractive agreements, but to assess several models consistently across an entire class of materials.

Much of the computational preparation would take place before beamtime. The participating groups would submit versioned models and calculate the observables expected for every sample, with blind-challenge predictions locked before release of the corresponding measurements. Predicted diffraction patterns and INS spectra would include the relevant scattering weights, temperature, isotopic composition and instrument response. During the experiment, an automatic sample changer would move through the library, data would be reduced through a common pipeline and measured signals would be compared rapidly with the precomputed predictions. Automated quality checks could flag an empty cell, misplaced sample or poor measurement, while larger scientific discrepancies would be retained for expert interpretation. The computing burden for forward simulation would usually be distributed before the experiment and is modest relative to training universal models, but data handling, sample preparation, quality control and scientific interpretation require dedicated staff. A campaign of this scale therefore needs coordinated contributions from sample providers, modeling groups, instrument scientists and research-software/data specialists rather than treating beamtime as the only scarce resource.

Powder diffraction provides a rapid structural test across crystalline material libraries. Lattice parameters, symmetry, phase purity and changes in average structure can be compared with model predictions using a consistent protocol. Low-temperature INS provides a complementary dynamical test. It is not restricted by optical selection rules, and its intensity can be calculated from patterns of atomic displacement using neutron scattering weights. The full spectrum therefore tests both vibrational energies and mode character. Critically, this technique probes the local energy landscape of the system, whose accuracy is precisely what determines the evolution of an MLIP. Low-temperature INS is nevertheless primarily a stringent near-equilibrium test; success there does not by itself establish high-temperature anharmonic behavior, activated transport or phase-transition kinetics, which is one reason for the complementary parametric layer. AbINS already converts normal modes from several electronic-structure packages and MLIPs into instrument-aware spectra,^14^ universal MLIPs have been assessed for rapid interpretation of INS data,^15^ and end-to-end workflows now connect DFT, MLIPs, molecular dynamics and simulated neutron observables.^16^ Many of the technical foundations for automated experiment–simulation comparison therefore already exist, although integrating them into a common validation program remains a substantial organizational and software task.

The result of the workhorse campaign ultimately would be a performance map for different MLIPs across many classes of materials, rather than a single leaderboard. A number summarizing the overall difference between prediction and measurement can help screen hundreds of cases, but it should not hide where disagreement occurs. The campaign should predeclare observable-specific metrics and uncertainty treatment. Diffraction might use errors in lattice parameters or volume, phase/symmetry agreement and profile residuals; INS could use peak-position errors, intensity or spectral-similarity measures and structured residuals; QENS could compare *S*(*Q*, *ω*) directly as well as derived quantities such as diffusion coefficients or residence times; parametric tests should additionally score slopes, shifts and transition points. Thresholds should be scientifically motivated for each observable and instrument rather than imposed as one universal numerical boundary. Patterns across the library are especially informative. Does one model struggle with flexible frameworks, another with hydrogen-rich guests and all models with a particular bonding environment? Because the simulated observables were prepared in advance and every sample followed the same protocol, the comparison would expose systematic strengths and weaknesses that isolated publications rarely reveal.

Parametric studies provide the complementary depth. Here, a small subset of chosen systems would each be examined at several state points, so the number of individual measurements could still be substantial. The systems might be chosen because they are scientifically important, because several models make distinguishable predictions or because failure is thought likely. Multiple temperatures could test phase behavior or diffusion, pressure could reshape intermolecular interactions/energy landscapes,^17–19^ and controlled dosing could follow adsorption and confinement.^20^ The central question is not simply whether the model agrees at one point. It is whether it reproduces the trend of how the material changes across thermodynamic space.

QENS and neutron total scattering sit naturally in this targeted mode. High-quality QENS data across multiple temperatures or loadings can require long measurements, careful resolution treatment and detailed analysis. What QENS can reveal also depends on the instrument’s energy and momentum-transfer windows and whether overlapping motions can genuinely be distinguished. A fair comparison applies the same resolution, ranges, assumptions and analytical models to experimental and simulated *S*(*Q*, *ω*). These restrictions on energy and momentum-transfer ranges further exemplify why such a campaign needs to purposely target specific systems and temperature ranges where information content is richest. Bayesian analysis can then compare possible mechanisms and quantities such as diffusion coefficients, residence times, jump lengths or rotational anisotropy.^21,22^ Simulation-informed analysis of liquid benzene illustrates how model predictions can recover dynamical information that conventional fitting could not identify uniquely.^23^

Total scattering can reveal changes in local structure that average diffraction misses, but collecting sufficiently precise data across several environments, isotope contrasts, and reducing them consistently is more demanding than routine diffraction. It is therefore most powerful when focused on a small number of systems for which local disorder, adsorption sites or short-range correlations are central to the model claim. Diffraction and INS can also operate in the parametric program. Their workhorse role does not prevent them from following a selected material across pressure, temperature or loading when the physical response is especially revealing.

A pressure-dependent broadband INS study of crystalline 2,5-diiodothiophene shows the value of this deeper test. The MLIP was challenged to reproduce both conventional blue shifts and an anomalous red shift associated with changing intermolecular interactions.^24^ Capturing these opposing responses is significant because compression does not simply stiffen every vibration. Although the MLIP does not model electrons explicitly, it is trained on quantum-mechanical calculations, so electronic effects are folded into its learned many-body potential. Reproducing the pressure dependence therefore provides evidence that this implicit electronic response evolves correctly within the conditions examined. Additionally, isotopic substitution can offer useful contrast or emphasize selected motions,^25^ and can be employed in all these techniques to extract additional information.

## Porous Materials are a Great Place to Start

Porous materials containing adsorbed or confined molecules provide an especially demanding test bed for MLIPs because they combine enormous chemical diversity with a rich and tunable physical response. The model must describe both the porous host and the molecular guest, the interactions between chemically distinct materials, and the way in which confinement reshapes intermolecular bonding, diffusion, and, where relevant, reaction barriers.^26^ Few systems challenge so many aspects of an interatomic potential at once, making successful predictions across these coupled behaviors particularly compelling evidence that the underlying physics has been captured.

A workhorse library could contain a large array of zeolites, metal–organic frameworks and related porous solids spanning framework topology, composition, guest identity and loading. Small organic guests could probe confinement and hydrogen bonding, while empty forms would provide controlled comparisons. The TOSCA broad band neutron spectrometer at the ISIS Neutron and Muon Source, UK^27,28^ offers a strong platform for the INS component because its spectra can be calculated directly from atomistic models and its established automatic sample changing allows prepared samples to be measured systematically at low temperature.^29^ Rapid powder diffraction could provide the corresponding structural benchmark.

Within the same program, a smaller subset could be explored in depth. Small organics confined in zeolites could be followed across guest loading or temperature.^28,30^ Hydrogen or carbon dioxide in metal–organic frameworks and porous carbons could test adsorption sites,^31,32^ framework response and molecular mobility. QENS could quantify diffusion or rotational motion, total scattering could reveal changes in local structure, and selected diffraction or INS measurements could track phase behavior and vibrational interactions. Combining techniques is particularly valuable because a model that reproduces average structure may still fail for dynamics, while an apparently correct spectrum may conceal the wrong local arrangement.

The workhorse and parametric campaigns would also support model improvement in different ways. The broad library would reveal recurring failure classes and poorly represented chemistries. The deeper studies would show which interaction, state point or mechanism breaks down. Developers could then add targeted reference calculations, alter training coverage or refine models using experimental information. New predictions would follow naturally, but they should be assessed against fresh held-out measurements so that development evidence is not recycled as validation. The purpose is not to maintain a permanent wall between measurement and development, but to ensure that the evidence attached to each claim remains clear. Experiment should be allowed to teach the model, while blind experiments remain available to test what the revised model has learned.

For that teaching to be reliable, the chain connecting sample, measurement and prediction must remain intact. A developer should be able to link a physical object and its history to the measured signal and the corresponding simulated observable without reconstructing the experiment from notebooks and local memory. Identity, composition, synthesis, batch, mass, geometry, containment and environmental history are part of the scientific evidence, not administrative decoration. The data-only illusion is especially dangerous when a spectrum becomes detached from the material that produced it.^33^ Raw counts, reduction history, calibration, resolution, backgrounds, uncertainty, model versions, simulation protocols and software versions should remain traceable. NeXus and FAIR-data programs provide an important foundation for these practices,^34,35^ while resources such as OpenKIM illustrate how persistent model identifiers, standardized metadata and verification tests can support reproducibility.^36^ Benchmark releases should include the experimental uncertainty model, machine-readable metadata and the exact model/workflow versions used for each prediction. Negative results and failed challenge cases should be published alongside successful ones because they are often the most informative data for future model development.

The required automation need not be elaborate. A dependable sample changer, robust identity tracking and a fixed reduction pipeline may be more valuable than a sophisticated closed loop built on uncertain data. Automatic comparison would accelerate quality control and reveal where prediction and measurement diverge, but scientific interpretation would remain a human responsibility. Parametric experiments would generally require more direct involvement from instrument scientists and sample-environment teams because each state change introduces additional controls and potential artifacts. In both modes, automation should strengthen experimental judgement rather than attempt to replace it.

The proposal also builds on, rather than replaces, several emerging initiatives. Matbench Discovery, MLIP Arena and UniFFBench already provide common computational or experimentally anchored frameworks for cross-model benchmarking.^4,7,37^ At large-scale facilities, automated sample handling and high-throughput end stations are also becoming established: robotic integration at NSLS-II, sample changers on neutron chopper spectrometers at the Spallation Neutron Source and high-throughput robotic GIWAXS at the Advanced Light Source demonstrate important parts of the required infrastructure.^38–40^ What is missing is the coordinated layer proposed here: reusable physical sample libraries, instrument-level forward predictions from versioned MLIPs, common uncertainty-aware protocols, and genuinely blind challenge measurements accumulated across model generations. The novelty is therefore less a new piece of hardware than a new way of organizing experimental validation around existing and rapidly developing capabilities.

## Campaign Science Needs Campaign Access

Campaigns of this scale do not fit comfortably within the conventional proposal built around one principal investigator, one instrument and a few days of beamtime. A workhorse library of order 10^2^ reusable samples, potentially extending to several hundred in a highly standardized campaign, together with 5–20 parametric systems, precomputed simulated observables and coordinated access to several instruments, already forms one substantial scientific work package. Across repeated campaigns, state points and successive versions of several models, the resulting resource can grow to thousands of comparable validation records without requiring thousands of new syntheses for every model generation. Fragmenting the package into unrelated proposals would weaken the common standards and comparability of the results, as well as making the outcome dependent on several independent allocation decisions. A program-access or consortium-led approach would allow the complete campaign to be assessed as one scientific endeavor and then scheduled across the instruments and cycles needed to deliver it.

This would extend rather than bypass traditional peer review. A consortium would submit a coherent scientific case defining the material class, model questions, sample library, measurement plan, simulations, uncertainty treatment and intended outputs. Facility review would still assess scientific importance, technical feasibility, safety, sample quality and the efficient use of beamtime. The difference is scale and coordination. Reviewers would judge whether the complete breadth-and-depth program can produce a decisive advance, rather than asking each instrument proposal to stand alone. The review process may even feed back into the proposed campaign, to ensure a more robust testing of models, and therefore would benefit from AI specialist on the panel alongside other specialists. For long term multi-year campaigns, block and long-term access mechanisms at major facilities provide useful precedents for sustained programs.

The consortium would bring together MLIP developers, materials chemists, sample providers/synthesizers, neutron-scattering experts, sample-environment engineers, research-software specialists and scientists working on statistics and uncertainty. Facility scientists should participate as research partners from the beginning, helping to identify which measurements are feasible and which can genuinely distinguish between competing physical descriptions. Crucially, no single group is likely to possess the ability to produce samples in mass, technical modeling expertise, software infrastructure and instrument knowledge needed for a campaign of this size. The collaboration is therefore not simply convenient; it is an essential part of the scientific design.

## AI Investment Must Reach Experiment

Funding should be assembled with the same breadth. National and international AI programs, materials and net-zero initiatives, industrial partnerships, mission-led research and training programs could support sample preparation, researchers, automation, software, computing and data stewardship, and even the purchase of beamtime. The UK AI for Science Strategy already recognizes high-value datasets, interdisciplinary teams, automation and responsive national facilities as enabling priorities.^41^ Experimental assessment should become a funded component of materials AI rather than an unfunded expectation placed on facility core budgets. Partnership arrangements should also recognize the additional staff effort and operational capacity required to deliver unusually large campaigns. In this way, investment attracted by AI could help sustain the experimental infrastructure that makes its predictions scientifically useful.

Figure 2 brings these elements together. Materials and modeling groups first assemble the sample library and precompute the simulated observables, while facility teams establish measurement protocols, automation and feasibility. Funding from AI, materials, industrial and international sources is combined around one consortium-led program. The resulting proposal enters established scientific and technical review before workhorse and parametric allocations are scheduled as connected parts of the same campaign. Measurements then produce both a broad performance map and detailed physical understanding. Some measurements enter model development, while designated blind challenge sets remain untouched until predictions are frozen; subsequent campaigns can then test the next generation of models on fresh evidence. Improving the reliability of MLIPs in this way could strengthen their use in screening and mechanistic studies across pharmaceuticals, energy materials and catalysis without overstating the confidence supported by any single benchmark.

**Figure 2:**
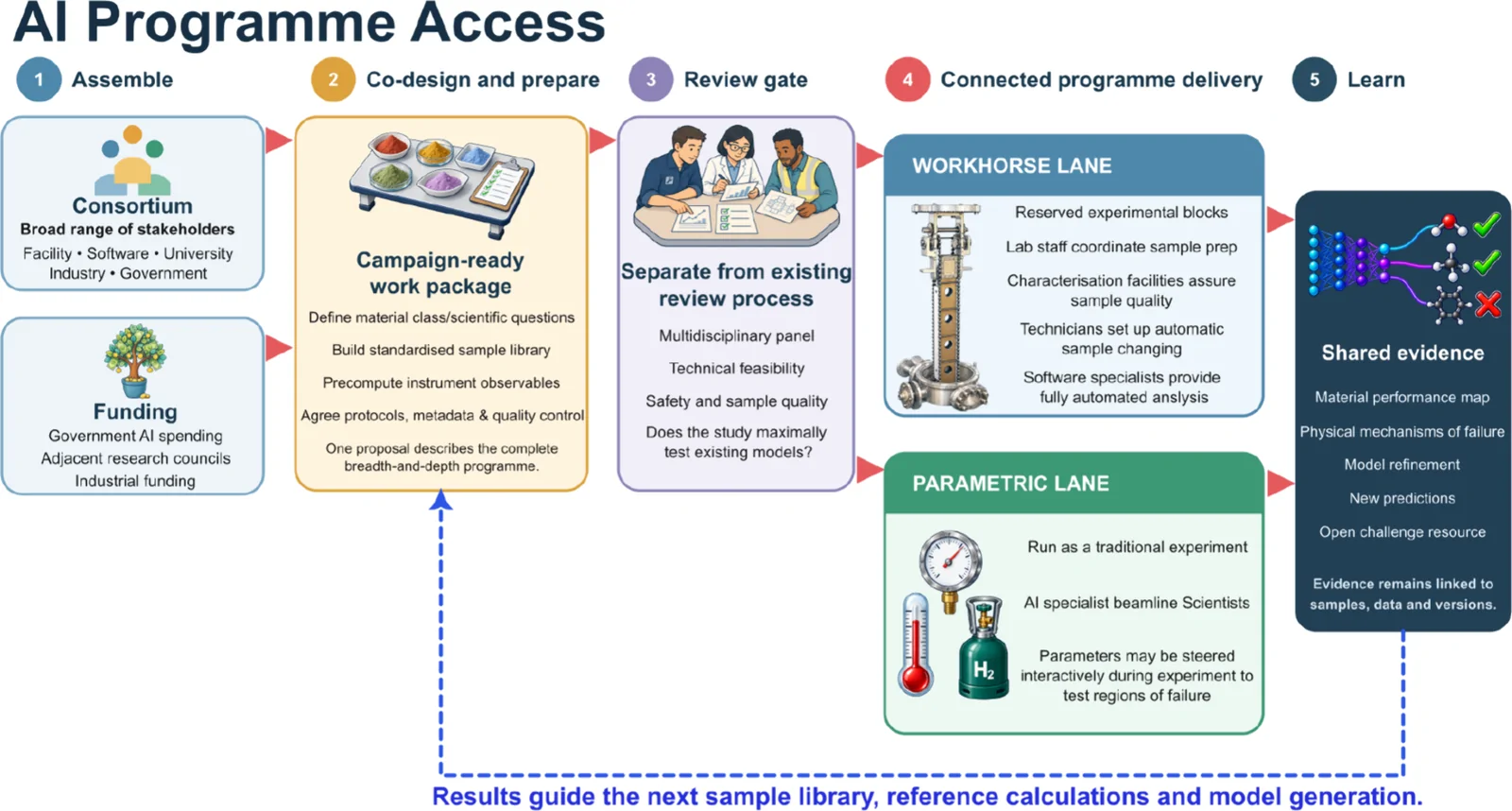
Proposed consortium led campaign workflow.

The opportunity for neutron facilities in the AI era is larger than supplying basic training data or automating routine measurements. They have the capabilities to assess atomistic model-and-simulation workflows across reusable libraries of real materials and then probe the most important systems deeply enough to understand why predictions succeed or fail. Doing so would turn isolated comparisons into a cumulative scientific resource and make experiment an active partner in model development. Facilities are not competing with AI for relevance. They provide physical evidence, skilled people and controlled environments through which predictions can earn bounded confidence. An AI strategy that funds prediction but not the capacity to measure, challenge and improve it is incomplete. AI can predict at a scale that experiment cannot match, but experiment is what keeps those predictions answerable to the physical world.

## Data Availability

No primary research data, software or code were generated for this Viewpoint.

## References

[1] Unke O. T., Chmiela S., Sauceda H. E., Gastegger M., Poltavsky I., Schütt K. T., Tkatchenko A., Müller K.-R. (2021). Machine learning force fields. Chem. Rev..

[2] Morrow J. D., Gardner J. L. A., Deringer V. L. (2023). How to validate machine-learned interatomic
potentials. J. Chem. Phys..

[3] Morrow J. D., Deringer V. L. (2022). Indirect learning and physically guided validation
of interatomic potential models. J. Chem. Phys..

[4] Riebesell J., Goodall R. E. A., Benner P., Chiang Y., Deng B., Ceder G., Asta M., Lee A. A., Jain A., Persson K. A. (2025). A Framework to Evaluate
Machine Learning Crystal Stability
Predictions. Nature Machine Intelligence.

[5] Deng B., Choi Y., Zhong P., Riebesell J., Anand S., Li Z., Jun K., Persson K. A., Ceder G. (2025). Systematic softening in universal
machine learning interatomic potentials. npj
Comput. Mater..

[6] Maxson T., Soyemi A., Zhang X., Chen B. W. J., Szilvási T. (2025). MS25: materials
science-focused benchmark data set for machine learning interatomic
potentials. J. Chem. Inf. Model..

[7] Mannan S., Bihani V., Gonzales C., Lee K. L. K., Gosvami N. N., Ranu S., Miret S., Krishnan N. M. A. (2026). UniFFBench: evaluating
universal machine learning force fields against experimental measurements. Nat. Comput. Sci..

[8] Schran C., Brezina K., Marsalek O. (2020). Committee neural network
potentials
control generalization errors and enable active learning. J. Chem. Phys..

[9] Vandermause J., Torrisi S. B., Batzner S., Xie Y., Sun L., Kolpak A. M., Kozinsky B. (2020). On-the-fly active learning of interpretable
Bayesian force fields for atomistic rare events. npj Comput. Mater..

[10] Zhang Y., Wang H., Chen W., Zeng J., Zhang L., Wang H., E W. (2020). DP-GEN: a
concurrent learning platform
for the generation of reliable deep learning based potential energy
models. Comput. Phys. Commun..

[11] Shanks B. L., Potoff J. J., Hoepfner M. P. (2022). Transferable
force fields from experimental
scattering data with machine learning assisted structure refinement. J. Phys. Chem. Lett..

[12] Thaler S., Zavadlav J. (2021). Learning neural network potentials
from experimental
data via differentiable trajectory reweighting. Nat. Commun..

[13] Han B., Yu K. (2025). Refining potential
energy surface through dynamical properties via
differentiable molecular simulation. Nat. Commun..

[14] Dymkowski K., Parker S. F., Fernandez-Alonso F., Mukhopadhyay S. (2018). AbINS: the
modern software for INS interpretation. Physica
B: Condensed Matter.

[15] Han B., Cheng Y. (2025). Benchmarking universal machine learning interatomic
potentials for
rapid analysis of inelastic neutron scattering data. Machine Learning: Science and Technology.

[16] Lindgren E., Jackson A. J., Fransson E., Berger E., Škoro G., Rudić S., Turanyi R., Mukhopadhyay S., Erhart P. (2025). Predicting neutron experiments from first principles:
a workflow powered by machine learning. J. Mater.
Chem. A.

[17] Armstrong J., Wang X., Fernandez-Alonso F. (2022). The unlocking of high-pressure science
with broadband neutron spectroscopy at the ISIS Pulsed Neutron &
Muon Source. Nucl. Instrum. Methods Phys. Res.,
Sect. A.

[18] Marin-Villa P., Gila-Herranz P., Jimenez-Ruiz M., Ivanov A., Armstrong J., Drużbicki K., Fernandez-Alonso F. (2025). Molecular Derailment via Pressurization
in Methylammonium Lead Iodide. J. Phys. Chem.
Lett..

[19] Marin-Villa P., Gaboardi M., Joseph B., Alabarse F., Armstrong J., Drużbicki K., Fernandez-Alonso F. (2025). Methylammonium Lead Iodide across
Physical Space: Phase Boundaries and Structural Collapse. J. Phys. Chem. Lett..

[20] Tian M., Lennox M. J., O’Malley A. J., Porter A. J., Krüner B., Rudić S., Mays T. J., Düren T., Presser V., Terry L. R. (2021). Effect of pore geometry
on ultra-densified hydrogen in microporous carbons. Carbon.

[21] Sivia D. S., Carlile C. J., Howells W. S., König S. (1992). Bayesian analysis
of quasielastic neutron scattering data. Physica
B: Condensed Matter.

[22] Omojola T., Silverwood I. P., O’Malley A. J. (2020). Molecular
behaviour of methanol and
dimethyl ether in H-ZSM-5 catalysts as a function of Si/Al ratio:
a quasielastic neutron scattering study. Catal.
Sci. Technol..

[23] Richardson H., McColl K., Nilsen G. J., Armstrong J., McCluskey A. R. (2026). Lost in
Translation: Simulation-Informed Bayesian Inference
Improves Characterization of Molecular Motion from Neutron Scattering. J. Phys. Chem. Lett..

[24] Armstrong J., Jackson A. J., Elena A. M. (2026). High-pressure inelastic
neutron spectroscopy:
experimental validation of machine-learned interatomic potential energy
landscapes. J. Phys. Chem. Lett..

[25] Armstrong J., Cavaye H., Sharma P., Potter M. E. (2026). Collective phonon
mixing and eigenvector transport under isotope substitution. Phys. Rev. B.

[26] Armstrong J., Banerjee S., Schünemann V., Wolny J. A., Sadler P. J. (2021). Vibrational
Motions Make Significant Contributions to Sequential Methyl C–H
Activations in an Organometallic Complex. J.
Phys. Chem. Lett..

[27] Pinna R. S., Rudić S., Parker S. F., Armstrong J., Zanetti M., Škoro G., Waller S. P., Zacek D., Smith C. A., Capstick M. J. (2018). The neutron guide upgrade
of the TOSCA spectrometer. Nucl. Instrum. Methods
Phys. Res., Sect. A.

[28] Morton K. S. C., O’Malley A. J., Armstrong J. (2025). Probing adsorption
interactions of lignin derivatives in industrial zeolite catalysts
through combining vibrational spectroscopy and ab initio calculations. RSC Sustainability.

[29] Parker S. F., Fernandez-Alonso F., Ramirez-Cuesta A. J., Tomkinson J., Rudic S., Pinna R. S., Gorini G., Castañon J. F. (2014). Recent
and future developments on TOSCA at ISIS. J.
Phys.:Conf. Ser..

[30] Morton K. S. C., Porter A. J., Armstrong J., O’Malley A. J. (2024). The effect
of molecular shape and pore structure on local and nanoscale cresol
behaviour in commercial zeolite catalysts. Catal.
Sci. Technol..

[31] Decarolis D., Ahmed S., King J., Elena A.-M., Zhang L., Armstrong J., Lezcano-Gonzalez I., Danaie M., Hirscher M., Meloni S. (2026). Functionalized MOFs for Subnanometric Control of Pd
Speciation for Selective Hydrogenation of Butadiene. ACS Appl. Nano Mater..

[32] Mor J., Sharma S. K., Bahadur J., Kancharlapalli S., Armstrong J. (2025). Fine-Tuning of Gate Opening in Hybrid Zeolitic Imidazolate
Framework-8: Inelastic Neutron Scattering and Density Functional Theory
Study. Langmuir.

[33] Smit B., Garcia S. (2026). The Data-Only Illusion
in Materials Discovery. Nat. Mater..

[34] Könnecke M., Akeroyd F. A., Bernstein H. J., Brewster A. S., Campbell S. I., Clausen B., Cottrell S., Hoffmann J. U., Jemian P. R., Männicke D. (2015). The NeXus data format. J. Appl. Crystallogr..

[35] Reeves-McLaren N. (2026). Raw data as
the verification layer: why AI-driven materials discovery needs experimental
infrastructure. ACS Omega.

[36] Tadmor E. B., Elliott R. S., Sethna J. P., Miller R. E., Becker C. A. (2011). The Potential
of Atomistic Simulations and the Knowledgebase of Interatomic Models. JOM.

[37] Chiang, Y. ; Kreiman, T. ; Zhang, C. ; Kuner, M. C. ; Weaver, E. J. ; Amin, I. ; Park, H. ; Lim, Y. ; Kim, J. ; Chrzan, D. MLIP Arena: Advancing Fairness and Transparency in Machine Learning Interatomic Potentials via an Open, Accessible Benchmark Platform. Advances in Neural Information Processing Systems, Datasets and Benchmarks Track. 2025; NeurIPS 2025 Spotlight.

[38] Fernando C., Marcello H., Wlodek J., Sinsheimer J., Olds D., Campbell S. I., Maffettone P. M. (2025). Robotic
integration for end-stations at scientific user facilities. Digital Discovery.

[39] Stone M. B., Granroth G. E., Pajerowski D. M., Abernathy D. L., Conner D. L., DeBeer-Schmitt L., Fanelli V. R., Goyette R., Kolesnikov A. I., Mills R. (2025). Sample changers for
direct geometry neutron chopper spectrometers. Sci. Rep..

[40] Schaible E., Galikeev I., Roizin-Prior M., Birkel G., Wang Y., Koepp W., Sriramoju R. V., Barnard H., Osubor C., Molsick-Gibson C. (2026). High-Throughput Robotic GIWAXS at ALS SAXS/WAXS
Beamline. Photon Sci..

[41] Department for Science, Innovation and Technology , AI for Science Strategy. https://www.gov.uk/government/publications/ai-for-science-strategy/ai-for-science-strategy, 2025 (accessed 17 July 2026).

